# The prevalence of turnover intention and influencing factors among emergency physicians: A national observation

**DOI:** 10.7189/jogh.12..04005

**Published:** 2022-02-05

**Authors:** Jie Feng, Liqing Li, Chao Wang, Pan Ke, Heng Jiang, Xiaoxv Yin, Zuxun Lu

**Affiliations:** 1 Department of Social Medicine and Health Management, School of Public Health, Tongji Medical College, Huazhong University of Science and Technology, Wuhan, China; 2 Department of Management Science and Engineering, School of Economics and Management, Jiangxi Science and Technology Normal University, Nanchang, Jiangxi, China; 3 Centre for Alcohol Policy Research, School of Psychology and Public Health, La Trobe University, Melbourne, Victoria, Australia; 4 Centre for Health Equity, Melbourne School of Population and Global Health, University of Melbourne, Melbourne, Victoria, Australia

## Abstract

**Background:**

High turnover among physicians in emergency department is a great challenge in China. However, the rate and the reasons why physicians intend to leave have not been extensively studied yet. Therefore, this study aimed to identify the risk factors of turnover intention among physicians in emergency department.

**Methods:**

A national cross-sectional survey was conducted from July to August 2018 in China. A total of 10 457 physicians in emergency department were investigated using a structured self-administered questionnaire, which included demographic characteristics, work-related factors, turnover intention, the Patient Health Questionnaire and Positive and Negative Affect Scale. The stepwise logistic regression analysis was applied to identify the risk factors of turnover intention among physicians.

**Results:**

A total of 55.18% of the physicians in emergency department reported turnover intention in China. Turnover intention were more likely to be reported among physicians who were male (odds ratio (OR) = 1.25, 95% confidence interval (CI)  = 1.13-1.38); who perceived medical errors (OR = 1.35, 95% CI = 1.23-1.47); who had a lower average monthly income; who provided out-of-hospital resuscitation services; who experienced physical violence (OR = 1.39, 95% CI = 1.26-1.54) and who reported higher score on negative affect and depressive tendency (OR = 1.06, 95% CI = 1.05-1.08; OR = 1.10, 95% CI = 1.09-1.12). Conversely, physicians who perceived shortage of manpower (OR = 0.74, 95% CI = 0.66-0.81), or reported higher score on positive affect (OR = 0.96, 95% CI = 0.94-0.97) were inclined to stay in emergency department.

**Conclusion:**

This study shows that turnover intention among physicians in emergency department is high in China and was significantly associated with gender, average monthly income, perceived shortage of physicians, the times for provide out-of-hospital resuscitation services, exposure to workplace violence, depressive tendency, positive affect and negative affect. Targeted interventions are required to improve the retention rate among physicians in emergency department from the comprehensive aspects.

The shortage of physicians in emergency department has been a growing global issue in recent years [1,2]. It is estimated that Canada will be shortfall of 1518 physicians in emergency department by 2025 [3]. According to a study in China, there was a deficiency in the regular supply of 2275 physicians in emergency department in Jiangsu province [4]. The phenomenon that numerous physicians working in emergency department quit their occupations, mainly affected by turnover intention, which is an important factor resulting in the insufficiency of emergency human resources.

Turnover intention, a psychological tendency to leave an organization or a position, could serve as an effective predictor of voluntary turnover behavior among employees [5-7], which has been widely adopted among physicians [8,9]. An integrative review containing 17 studies from five countries (the UK, Germany, the USA, France and Finland), showed that the percentage of physicians with turnover intention varied from 11.8% to 22% [10]. According to a study from Norway found that 21.0% of all hospital physicians expressed an intention to leave their current job [11]. The differences in social and economic development and the working environment of physicians may lead to the obvious diversities of the rate of the physicians’ turnover intention among various countries. At present, the studies on turnover intention mainly focused on physicians majoring in oncology, internal medicine, anesthesia, psychiatry and so on [8,9,12-14], while relevant empirical researches seemed scarce about turnover intention among physicians in emergency department. Working under highly stressful and fast-paced working environment may be the contributors for physicians intend to leave emergency department [15]. An international multi-centre survey conducted in 24 countries showed that 33.8% of the physicians in emergency department were more likely to quit their jobs [16]. Furthermore, the higher the turnover intention among the physicians in emergency department, the more likely they are inclined to take actions to quit their positions. The turnover of physicians will not only aggravate the shortage of emergency health human resources, but also could cause the insufficient supply of emergency medical services, thus obstructing people’s access to health care and endangering their lives and health [17]. Therefore, identifying the factors related to turnover intention is particularly vital for reducing the turnover rate of physicians in emergency department.

Up to now, studies investigating turnover intention among physicians in emergency department and its risk factors (such as demographic characteristics, job-related factors, job satisfaction and job burnout) have been conducted in many developed countries. [15,18] However, there have been few studies on the turnover intention among physicians in emergency department in China.[19,20] Considering the lack of research on the risk factors of turnover intention among health care providers in China, particularly in emergency department, a large-scale national survey among physicians in emergency department in China was conducted in this study, aiming to explore the factors related to turnover intention from the aspects of individual factors, work-related factors and internal psychological factors, which could provide data support and scientific basis for proposing effective interventions to decrease the turnover intention among physicians in emergency department.

## METHODS

### Ethics

Ethical approval for this study was obtained from the Medical Ethics Committee of Hainan Medical College (HYLL-2018-035). Before conducting the survey, we obtained the informed consent of participants, and each participant was voluntary. We promised the participants that any information they provided would be used solely for scientific research and that their personal information would be kept strictly confidential.

### Study design and sample selection

This study was part of a nationwide cross-sectional survey of Emergency Medical Resources conducted in 31 provinces of China, with the coordination of the Medical Administration Bureau of the National Health Commission of the People’s Republic of China, from July to August 2018. An anonymous questionnaire with standard structure was used to collect data through an online survey platform in China (platform name: the QuestionnaireStar program, URL: https://www.wjx.cn). The link of the web-based questionnaire was posted on the emergency physicians’ working platform of the pre-hospital emergency facility configuration monitoring department, inviting physicians in emergency department to participate anonymously in this cross-sectional online survey. Additionally, the electronic questionnaire was reposted to social networking sites every 7 days to remind physicians to complete it until the end of the survey. Physicians who completed the questionnaire by visiting the link were required to read and agree to the electronic informed consent statement before answering. All data were stored and managed uniformly by the QuestionnaireStar program (Changsha Ranxing Information Technology Ltd, Changsha, China).

### Quality control

Two main quality control measures were adopted in this study to guarantee the quality of the online questionnaire results. First, to prevent repeated submissions of questionnaires from the same participant, each mobile phone number could only be used once. Second, we set up three quality control questions at different places in the questionnaire to identify the participants who filled the questionnaire at random, including “Where is the capital of China?” and “What is 3 plus 5?” and “What is 7 minus 2?”. Each question had 4 alternative answers, of which only one was correct. The online questionnaire system would mark the questionnaire as invalid if incorrect answers to the quality control questions appeared.

### Instrument and measurement

The content of electronic questionnaire included the demographic characteristics of participants, work-related factors, turnover intention, the Patient Health Questionnaire, Positive and Negative Affect Scale. Specifically, the demographic characteristics of participants included age, gender, marital status, education level and self-perceived physical health, geographical region. Work-related factors included average monthly income, self-perceived shortage of physicians, the number of patients seen by the physicians per day, the times for physicians to provide out-of-hospital resuscitation services per week, self-perceived medical errors in the last three month, whether the physicians in emergency department experienced verbal violence in the past year and whether the physicians in emergency department experienced physical violence in the past year.

Turnover intention was measured by asking participants one question “I intend to quit my present job as soon as possible.” This item was answered on a 5-point Likert scale, ranging from 1 (strongly disagree), 2 (disagree), 3 (partly agree), 4 (agree) to 5 (strongly agree), with a score of 4 or 5 indicating intention to leave.

Depressive tendency was measured by the Patient Health Questionnaire (PHQ-9) [21], a self-reporting instrument with nine questions. The scores ranged from 0 (not at all), to 3 (nearly every day). The total score of depressive tendency ranges from 0 to 27. Higher scores indicate a higher level of depressive tendency. And the reliability for the scale was fairly strong as well (Cronbach’s α = 0.92).

Positive affect and negative affect were measured by the 10-item Positive and Negative Affect Scale (PANAS) [22], which comprised of two subscales: positive affect (5 items) and negative affect (5 items). Positive affect measures the degree to which a person is predisposed to experience positive emotions and moods, such as pleasure, enthusiasm and energy. While negative affect is defined as a trait that describes the tendency of an individual to experience a variety of negative emotions and moods, such as anger, guilt and tension [22]. The PANAS was carried out based on a 5-point Likert scale, ranging from 1 (strongly disagree) to 5 (strongly agree). Higher score reflects higher level of positive affect or negative affect, respectively. In our study, the positive affect (Cronbach’s α = 0.90) and negative affect (Cronbach’s α = 0.87) subscales have demonstrated high internal consistency.

### Statistical analysis

In this study, analyses were performed using SPSS version 23.0 (SPSS, Inc, Chicago, Illinois, USA). Descriptive analyses were performed to analyze characteristics among physicians. Specifically, means and standard deviations (SD) for continuous variables, frequencies and percentages for categorical variables were reported separately in our research. χ2 tests were conducted to compare the distribution of categorical variables between the group with turnover intention and the group without among physicians in emergency department. *t* tests were conducted to compare the positive affect score, the negative affect score, and the depression tendency score between groups with or without turnover intention. Binary logistic regression with a forward stepwise selection approach (level for selection: *P* = 0.05, and level for elimination: *P* = 0.10) was performed to examine the association of the independent variables with the turnover intention among physicians in emergency department, and adjusted odds ratios (ORs) and 95% confidence intervals (CI) for each variable were presented. Multicollinearity of the independent variables was tested by calculating the variance inflation factor (VIF) [23]. Finally, in sensitivity analysis, we excluded the participants within the lowest 10% in terms of response time to test the stability of the model. All statistical tests were two-tailed, and *P* value <0.05 was considered to be statistically significant.

## RESULTS

### Participants’ characteristics for socio-demographic variables, work-related factors, and internal psychological factors

A total of 15 288 physicians working in the emergency department clicked on the link of the questionnaire, and 10 457 physicians completed the questionnaire, with an overall response rate of 68.40%. Among them, 3780 came from eastern region, 2830 from the central region, and 3820 from the western region, accounting for 36.41%, 27.06% and 36.53% of the total sample respectively.

The main characteristics of the participants are reported in **Table 1****.** The mean age of total 10 457 participants was 36.53 (SD = 7.55) years. More than 70% participants were male, most of participants (84.42%) got married and nearly 70% obtained a bachelor degree. 36.61% of them reported themselves in poor physical condition. Moreover, 71.00% of the participants with an average monthly income of less than or equal to CNY 6000 (US$ 907.44), approximately half of the participants to rescue patients on the ambulance per week more than 11 times per week, 26.68% of them reported a shortage of physicians and almost half (43.63%) reported perceived medical errors during the previous three months prior to the survey. Overall, nearly one in third of participants have experienced physical violence from patients, and about 80% of them have suffered verbal violence. The mean score of positive affect, negative affect and depressive tendency for the physicians were 15.20 (SD = 3.99), 16.76 (SD = 3.96) and 8.92(SD = 5.80).

**Table 1:** Descriptive statistics for characteristic of the participants

Variables	All subjects		Turnover intention				*P*-value
**Yes**		**No**		
**n**	**%**	**n**	**%**	**n**	**%**	
**Total**	10 457	100	5770	55.18	4687	44.82	
**Individual factors:**							
Age:							<0.001
≤30	2600	24.86	1498	25.96	1102	23.51	
31-40	4977	47.59	2628	45.55	2349	50.12	
41-50	2388	22.84	1348	23.36	1040	22.19	
≥51	492	4.70	296	5.13	196	4.18	
Gender:							
Male	7632	72.98	4031	69.86	3601	76.83	<0.001
Female	2825	27.02	1739	30.14	1086	23.17	
Marital status:							
Unmarried/widowed/divorced/separated	1629	15.58	921	15.96	708	15.11	0.230
Married	8828	84.42	4849	84.04	3979	84.89	
Education level:							<0.001
Associated degree or less	1684	16.10	1022	17.71	662	14.12	
Bachelor degree	7789	74.49	4180	72.44	3609	77.00	
Master degree or higher	984	9.41	568	9.84	416	8.88	
Self-perceived physical health:							<0.001
Good	1499	14.33	1129	19.57	370	7.89	
Fair	5130	49.06	3077	53.33	2053	43.80	
Poor	3828	36.61	1564	27.11	2264	48.30	
Geographical region:							0.005
Eastern region	3807	36.41	2086	36.15	1721	36.72	
Central region	2830	27.06	1578	27.35	1252	26.71	
Western region	3820	36.53	2106	36.50	1714	36.57	
**Work-related factors:**							
Average monthly income (CNY):							<0.001
Less than or equal to 4000 (US$ 604.96)	3862	36.93	1982	34.35	1880	40.11	
4001-6000 (US$ 605.11-US$ 907.44)	3562	34.06	1976	34.25	1586	33.84	
6001-8000 (US$ 907.59-US$ 1209.92)	1904	18.21	1113	19.29	791	16.88	
8001 and above (US$ 1210.07 and above)	1129	10.80	699	12.11	430	9.17	
Self-perceived shortage of physicians:							<0.001
Yes	2790	26.68	1919	33.26	871	18.58	
No	7667	73.32	3851	66.74	3816	81.42	
The number of patients seen by the physicians (per day):							<0.001
0-10	4333	41.44	2475	42.89	1858	39.64	
11-20	2202	21.06	1189	20.61	1013	21.61	
21-30	1547	14.79	864	14.97	683	14.57	
31-40	776	7.42	422	7.31	354	7.55	
≥41	1599	15.29	820	14.21	779	16.62	
The times for physicians to provide out-of-hospital resuscitation services (per week):							<0.001
0	986	9.43	642	11.13	344	7.34	
1-10	5258	50.28	3068	53.17	2190	46.72	
11-20	2124	20.31	1096	18.99	1028	21.93	
21-30	1071	10.24	498	8.63	573	12.23	
≥31	1018	9.74	466	8.08	552	11.78	
Self-perceived medical errors:							<0.001
Yes	4562	43.63	2034	35.25	2528	53.94	
No	5895	56.37	3736	64.75	2159	46.06	
Experienced verbal violence in the past year:							<0.001
Yes	8555	81.81	4400	76.26	4155	88.65	
No	1902	18.19	1370	23.74	532	11.35	
Experienced physical violence in the past year:							<0.001
Yes	2889	27.63	1144	19.83	1745	37.23	
No	7568	72.37	4626	80.17	2942	62.77	
**Internal psychological factors:**							
Positive affect	15.20 ± 3.99		16.28 ± 3.67		13.87 ± 3.97		<0.001
Negative affect	16.76 ± 3.96		15.64 ± 3.80		18.13 ± 3.71		0.501
Depressive tendency	8.92 ± 5.80		6.88 ± 4.74		11.44 ± 6.01		<0.001

### Rates of turnover intention and its association with demographic characteristic, work-related factors, and internal psychological factors

**Table 1** demonstrated that 55.18% of the physicians intend to leave. It was suggested that there were differences in distributions between the group with turnover intention and the group without (*P* < 0.05), by age, gender, education level, average monthly income, geographical region, self-perceived physical health, self-perceived shortage of physicians, self-perceived medical errors, the number of patients seen by the physicians, the times for physicians to provide out-of-hospital resuscitation services and exposure to workplace violence, positive affect and depressive tendency.

### The associations between predictor variables and turnover intention

The results of the stepwise logistic regression analysis of turnover intention according to individual factors, work-related factors and internal psychological factors are displayed in **Table 2****.** Regarding individual factors, risk factor contributing to turnover intention was gender. Compared with female physicians, male had higher odds of reporting turnover intention (OR = 1.25, 95% CI = 1.13-1.38). Physicians, having lower average monthly income, not perceiving shortage of physicians, having to provide out-of-hospital resuscitation services, reporting self-perceived medical errors and having experienced physical violence were more likely to quit their position. Additionally, the turnover intention among physicians in emergency department is associated with a 1.06-unit increase in negative affect and 1.10-unit increase depressive tendency. Each 1-point increase in positive affect score was associated with a 4% decrease in the odds of the turnover intention among physicians in emergency department. The VIF, which assesses how much the standard error of model estimates is inflated due to multicollinearity, ranged from 1.08 to 3.30, well below the recommended cut-off value of 10. The multicollinearity diagnosis for this study was shown in Table S1 in the **Online Supplementary Document**.

**Table 2:** The stepwise logistic regression for the associations between predictor variables and turnover intention

Variables	Turnover intention	*P*-value
**Constant**		
**Individual factors:**		
Gender (Ref: Female):		
Male	1.25 (1.13-1.38)	<0.001
**Work-related factors:**		
Average monthly income (CNY) ((Ref: Less than or equal to 4000 (US$ 604.96)):		
4001-6000 (US$ 605.11-US$ 907.44)	0.82 (0.74-0.91)	<0.001
6001-8000 (US$ 907.59-US$ 1209.92)	0.76 (0.67-0.85)	<0.001
8001 and above (US$1210.07 and above)	0.72 (0.62-0.84)	<0.001
Self-perceived shortage of physicians (Ref: No):		
Yes	0.74 (0.66-0.81)	<0.001
The times for physicians to provide out-of-hospital resuscitation services (per week) (Ref: 0):		
1 -10	1.25 (1.07-1.47)	<0.001
11-20	1.41 (1.19-1.68)	0.005
21 -30	1.56 (1.28-1.91)	<0.001
≥31	1.50 (1.22-1.83)	<0.001
Self-perceived medical errors (Ref: No):		
Yes	1.35 (1.23-1.47)	<0.001
Experienced physical violence in the past year (Ref: No):		
Yes	1.39 (1.26-1.54)	<0.001
**Internal psychological factors:**		
Positive affect*	0.96 (0.94-0.97)	<0.001
Negative affect*	1.06 (1.05-1.08)	<0.001
Depressive tendency*	1.10 (1.09-1.12)	<0.001

*Parameter estimates indicate the change in each metric associated with turnover intention. For instance, the physicians’ turnover intention is associated with a 1.06-unit increase in negative affect.

### Sensitivity analysis

In this study, the primary results were not changed after excluding the participants within the lowest 10% in terms of response time. The sensitivity analysis for the association between predictor variables and turnover intention was shown in Table S2 in the **Online Supplementary Document**.

## DISCUSSION

This nationwide population-based cross-sectional study indicated that 55.18% of physicians, working in emergency department, intend to quit their position in China, which was obviously higher than that of an international multi-centre survey conducted in 24 countries (33.8%) [16] and another survey conducted in France (24%) [15]. This difference was possibly related to social environment, the level of economic development and the working environment among various countries. Also, due to the heavy workloads and working without adequate rest that the rate of the turnover intention among physicians in emergency department was much higher than that of radiologists (33.3%), general practitioners (47%) and physicians in intensive care (26%) [24-26].

There was a statistically significant difference in turnover intention between male and female among physicians in emergency department. We found that the intention to leave their current positions of male physicians was much stronger than female physicians. The main reason for the result was that, in the context of traditional Chinese culture, men are more liable to leave their present posts than their female colleagues because of stronger achievement motivation and adventurous spirit [27]. And similar results have been reported in Korea and Norway to explain the phenomenon that male physicians and nurses are more liable to leave present positions [28,29]. It was found that average monthly income was negatively related to turnover intention among physicians in emergency department. It is probably due to the lower average monthly income may not cover living needs for physicians in emergency department, so the physicians who have a lower average monthly income were more likely to leave emergency department, which was in line with previous study [30].

Working environment is essential to the improvement of physicians’ retention. First, physicians who admitted that they had made medical errors over the last three months were more likely to quit their jobs. Medical errors not only lead to increase numbers of adverse events, such as medication errors, failure to recognize life-threatening signs and symptoms, but also cause considerable damage to emotional health among physicians working in emergency department, including loss of self-esteem and confidence. And then it will significantly increase their turnover intention, which was in line with the results of existing researches [31,32]. In this study, physicians who provided out-of-hospital resuscitation services would positively have more intense incentive to depart form present occupations. Therefore, hospital administrators should take measures to reasonably arrange the times of physicians to rescue patients outside the hospital, so as to prevent the resignation among physicians. Moreover, the turnover intention of physicians who perceived shortage of manpower would be lower. The possible explanation is that, emergency department is a very important department for patients, therefore, with the strong sense of obligation and desire to fulfill professional mission well, the physicians who perceived the shortage of manpower were more likely to stay in emergency department to save the lives of critically ill patients by their endeavors [33]. Moreover, there was no such report in existing studies on the correlation between self-perceived shortage of manpower and turnover intention among physicians. The results obtained in the present study may provide an interesting finding. Furthermore, physicians who had been directly suffering from patient-initiated violence were found to have higher turnover intention compared to those who had never or less been exposed to, which corresponds to the results of the previous study [34-36]. Different from other departments, physicians in emergency department are very prone to undergo workplace violence, attributing to a fact that the patients who are seriously ill or injured will be accepted in this department, and the patients and their families are emotionally unstable [37]. Considering the urgent situation of patients, there is rare time for communication between physicians and patients. Therefore, it is suggested that hospital administrators should endeavor to ensure the security of physicians and strengthen interpersonal communication abilities of physicians, which may lessen the occurrence of similar violence for physicians.

It is noteworthy that internal psychological factors were significantly associated with turnover intention as well. Physicians with higher positive affect score shown fewer turnover intention, while with higher depressive tendency and negative affect scores were more likely to resign from their jobs. A possible explanation to this phenomenon is that, physicians with positive attitude are able to actively deal with various obstacles in emergency assignments and to feel satisfied with the results of their work, then are more willing to stay in emergency department. Conversely, high level of negative affect and depressive tendency make physicians full of complaints about their intensive assignments and regard works as a burden [38], which dramatically increases the possibility of turnover intention [39]. The above-mentioned findings demonstrate that it is also necessary to pay attention to the psychological environment of physicians apart from stable external working environment. Hospital administrators should take a series of measures, such as conducting psychological consultations, holding lectures about mental health and so on, to alleviate the negative emotions of physicians and stimulate their enthusiasm on emergency work, in order to reduce the negative impact of unhealthy intrinsic characteristics on turnover intention.

The strength of this study was that the study sample was well represented because the participants were balanced across the eastern, central, and western regions of China. And these three regions represented the economically developed, average, and less developed regions of China, respectively. Moreover, the numerous samples obviously increased statistical power to identify social factors of the turnover intention among physicians working in emergency department. It will play a role in providing a reference for related authorities to establish effective policies and increase the stability of physicians in emergency department. There are still several limitations in this study. First, our research is a cross-sectional study, so it is impossible to identify the causal relationship between turnover intention and related factors among physicians in emergency department, which requires a further verification by longitudinal studies. Second, most of information was relied on a self-reported questionnaire from physicians in emergency department, thus recall bias is hard to be avoided completely. Third, given that approximately 30% of the participants did not complete the survey, this study may be prone to response bias.

## CONCLUSIONS

In conclusion, turnover intention is common among physicians in emergency department and is significantly associated with the workplace environment (self-perceived shortage of physicians, the times for physicians to provide out-of-hospital resuscitation services, exposure to workplace violence) and certain psychological factors. Targeted interventions, such as take measures to preferably prevent and manage workplace violence and to decrease the level of depression tendency and negative affect and so on, are required to improve the retention rate among physicians in emergency department from the comprehensive aspects, including external working conditions and internal psychological environment.

## Additional material


Online Supplementary Document
