# Supplementary material for: The prevalence of turnover intention and influencing factors among emergency physicians: A national observation
Source: J Glob Health. 2022 Feb 5;12:04005. doi: 10.7189/jogh.12..04005 (PMC8818293; doi:10.7189/jogh.12..04005)
Supplement: Online Supplementary Document [file jogh-12-04005-s001.pdf]

Online Supplement Document

Table S1. The Multicollinearity test results

Variables	Tolerance	Variance inflation factor
**Total**		
Individual factors		
**Age**	0.78	1.27
**Gender(Ref: Female)**		
Male	0.92	1.08
**Marital status(Ref: Unmarried/widowed/divorced/separated)**		
Married	0.86	1.16
**Education level(Ref: Associated degree or less)**		
Bachelor degree	0.63	1.58
Master degree or higher	0.62	1.61
**Self-perceived physical health(Ref: Good)**		
Fair	0.39	2.55
Poor	0.30	3.30
**Geographical region(Ref: Eastern region)**		
Central region	0.75	1.34
Western region	0.75	1.33
Work-related factors		
**Average monthly income(CNY) ((Ref: Less than or equal to 4000(US$604.96))**		
4001-6000(US$605.11-US$907.44)	0.74	1.35
6001-8000(US$907.59-US$1209.92)	0.74	1.35
8001 and above(US$1210.07 and above)	0.75	1.33
**Self-perceived shortage of physicians(Ref: No)**		
yes	0.87	1.16
**The number of patients seen by the physicians (per day) (Ref: 0~10)**		
11~20	0.81	1.23
21~30	0.82	1.22
31~40	0.87	1.15
≥41	0.77	1.29
**The times for physicians to provide out-of-hospital resuscitation services (per week) (Ref: 0)**		
1~10	0.31	3.22
11~20	0.38	2.62
21~30	0.51	1.97
≥31	0.52	1.94
**Self-perceived medical errors(Ref: No)**		
yes	0.87	1.15
**Experienced verbal violence in the past year (Ref: No)**		
yes	0.80	1.24

**Experienced physical violence in the past year (Ref: No)**		
yes	0.84	1.19
Internal psychological factors		
**Positive affect**	0.57	1.76
**Negative affect**	0.59	1.68
**Depressive tendency**	0.45	2.22

Table S2. The sensitivity analysis for the association between predictor variables and turnover intention

Variables	Turnover intention	*P*-value
**Constant**		
Individual factors		
**Age**	1.01(1.00-1.02)	0.007
**Gender (Ref: Female)**		
Male	1.29(1.16-1.44)	<0.001
Work-related factors		
**Average monthly income(CNY) ((Ref: Less than or equal to 4000(US$604.96))**		
4001-6000(US$605.11-US$907.44)	0.82(0.73-0.91)	<0.001
6001-8000(US$907.59-US$1209.92)	0.75(0.66-0.85)	<0.001
8001 and above(US$1210.07 and above)	0.69(0.59-0.81)	<0.001
**Shortage of physicians(Ref: No)**		
Yes	0.65(0.58-0.73)	<0.001
**The times for physicians to provide out-of-hospital resuscitation services (per week) (Ref: 0)**		
21~30	1.32(1.13-1.53)	<0.001
≥31	1.25(1.07-1.44)	0.005
**Self-perceived medical errors(Ref: No)**		
Yes	1.26(1.15-1.39)	<0.001
**Experienced verbal violence in the past year (Ref: No)**		
Yes	1.17(1.02-1.34)	0.029
**Experienced physical violence in the past year (Ref: No)**		
Yes	1.35(1.21-1.50)	<0.001
Internal psychological factors		
**Positive affect***	0.94(0.93-0.96)	<0.001
**Negative affect***	1.05(1.03-1.06)	<0.001
**Depressive tendency***	1.11(1.10-1.12)	<0.001

Exclude the participants within the lowest 10% in terms of response time.

*Parameter estimates indicate the change in each metric associated with turnover intention. For instance, the physicians' turnover intention is associated with a 1.05-unit increase in negative affect.
